# Distribution of Population by Ages

**Published:** 1913-10

**Authors:** 


### Distribution of Population by Ages

Medical articles dealing with the incidence of diseased conditions frequently contain false statements or implications, due to neglect to consider the number of persons of any age group, to whom the law of chance applies. In any ordinary community the sexes are nearly equal though females exceed males at birth (more sown of the better crop), slightly throughout life and considerably as age advances. The following statistics are based, respectively, on reports of about 30,000,000 in registration areas, taking the population as it runs, which, on account of immigration, exaggerates the number of males and active adults, and on a similar series of about 3,000,000 born of native white parents:

| Age. | % native born of native born. | Total to age limit. | % whole population | Total to age limit |
|---|---|---|---|---|
| Under 1 yr. | 2.4 | | 2.1 | |
| 1-4 | 8.8 | 11.2 | 8.1 | 10.2 |
| 5-14 | 20.6 | 31.8 | 19. | 29.2 |
| 15-24 | 18.2 | 50. | 18.9 | 48.1 |
| 25-34 | 15.1 | 65.1 | 18.2 | 66.3 |
| 35-44 | 11.9 | 77. | 14. | 80.3 |
| 45-64 | 16.4 | 93.4 | 15. | 95.3 |
| Over 65 | 6.2 | 99.6 | 4.4 | 99.7 |
| Error due to unknown and estimating fractions | .4 | 100. | .3 | 100. |

Note that the first age period given is for a single year, the second for a four-year period, the next to the last for a double decade and the last for a span of about forty years. With these allowances, it is apparent that the number of persons living steadily decreases as the age-period is advanced. This is due not only to the cumulative mortality but to the fact that each decennial period represents an aggregate population of about 10 per cent. more than the next older, on account of the excess of births.

For example, a disease with no special age incidence, would have its mortality almost equally divided between those under and those over 25; so, too, about half of its mortality would fall between the ages of 20 and 50. On the other hand, the statement that the susceptibility is very slight in the aged, loses much of its significance when we consider that less than 5 per cent. are over 65. It must be remembered, also, that for many diseases, especially the semelincident, exanthematous infections, the actual greater incidence and mortality at early ages need not indicate any special susceptibility according to age. By the law of chance the majority of persons will be exposed fairly early in life and will, thereafter, be immune.

Let us test the general idea that tuberculosis is especially a disease of adolescence and early adult life and see how it works out. Statistics of 1900 will be used, simply because the Census Bureau has not had a sufficient appropriation to publish the 1910 figures.

Of 109,492 total deaths from consumption, 8,036 (less than 8 per cent.) occurred before the age of 15, whereas nearly 30

per cent. of the population are included in this period. From 15-19, a little over 9,084 succumbed, corresponding very closely with the average mortality to be expected. From 20 to 24, nearly 16,000 succumbed, showing a mortality about 1½ what would be expected for an average incidence with special age susceptibility. From this point on there is a very gradual relative decrease, but it remains about 1 1/3 the average on the hypothesis of an equal susceptibility at all ages, as late as the 20-year period 45-64, and is fully as high for advanced ages. In other words, for some reason or other, there is an actual escape from consumption up to adolescence, a relative increase reaching almost exactly the theoretic average for adolescence, and a very slight relative predisposition for early adult life, probably no more than is to be accounted for by the law of chance in infection, as stated for the semelincident diseases.

For cancer, the general idea is correct, but its full force is scarcely realized. The cancer mortality is very small (about 1 1/3 per cent. of the total cancer deaths) up to the twentieth year. For the next three quinquenniums it progresses by geometric series, by 2, but is still small. Up to the twenty-fifth year it is a trifle over 2 per cent. of the total, although the population up to this age amounts to over 48 per cent. of the total. The cancer deaths increase actually, from quinquennium to quinquennium, in spite of the fact that the population of corresponding ages is diminishing up to the sixty-fifth year. Forty-five per cent. of all cancer deaths occur in the 20-year period between 45 and 64, comprising only 15 per cent. of the population. While, after this, the actual numbers by quinquenniums, decrease, the proportionate mortality continues to increase so that 35 per cent. of all cancer deaths occur in a population over 65, amounting to less than 5 per cent. of the total.

Looking at the matter from a different standpoint, we get somewhat different results. Under 20, about 1 death in a thousand occurs from cancer; from 20 to 24, 1 in 250; from 25 to 34, 1 in 77; from 35 to 44, 1 in 24; from 45 to 54, 1 in 11; from 55 to 64, 1 in 12; over 65, 1 in 20. The discrepancy is easily explained. From the standpoint of the living population, the tendency to death from cancer constantly increases, but, as age advances, so many trivial causes may result fatally, that the actual chance of dying of cancer is relatively lessened. Probably, if exact statistics were available, it would actually be shown that a great many old persons, dying of pneumonia, etc., actually were cancerous.

It would be interesting to continue this study further, but enough has been said to illustrate the importance of considering how many persons of any age group there may be, before jumping at conclusions regarding special tendencies to death from any given disease.

We append the American table of mortality:

| Age | Number Living | Number Dying Each Year | Age | Number Living | Number Dying Each Year |
|---|---|---|---|---|---|
| 10 ....... | 100,000 | 749 | 53....... | 66,797 | 1,091 |
| 11 ....... | 99,251 | 746 | 54....... | 65,706 | 1,143 |
| 12 ....... | 98,505 | 743 | 55....... | 64,563 | 1,199 |
| 13 ....... | 97,762 | 740 | 56....... | 63,364 | 1,260 |
| 14 ....... | 97,022 | 737 | 57....... | 62,104 | 1,325 |
| 15 ....... | 96,285 | 735 | 58....... | 60,779 | 1,394 |
| 16 ....... | 95,550 | 732 | 59....... | 59,385 | 1,468 |
| 17 ....... | 94,818 | 729 | 60....... | 57,917 | 1,546 |
| 18 ....... | 94,089 | 727 | 61....... | 56,371 | 1,628 |
| 19 ....... | 93,362 | 725 | 62....... | 54,743 | 1,713 |
| 20 ....... | 92,637 | 723 | 63....... | 53,030 | 1,800 |
| 21 ....... | 91,914 | 722 | 64....... | 51,230 | 1,889 |
| 22 ....... | 91,192 | 721 | 65....... | 49,341 | 1,980 |
| 23 ....... | 90,471 | 720 | 66....... | 47,361 | 2,070 |
| 24 ....... | 89,751 | 719 | 67....... | 45,291 | 2,158 |
| 25 ....... | 89,032 | 718 | 68....... | 43,133 | 2,243 |
| 26 ....... | 88,314 | 718 | 69....... | 40,890 | 2,321 |
| 27 ....... | 87,596 | 718 | 70....... | 38,569 | 2,391 |
| 28 ....... | 86,878 | 718 | 71....... | 36,178 | 2,448 |
| 29 ....... | 86,160 | 719 | 72....... | 33,730 | 2,487 |
| 30 ....... | 85,441 | 720 | 73....... | 31,243 | 2,505 |
| 31 ....... | 84,721 | 721 | 74....... | 28,738 | 2,501 |
| 32 ....... | 84,000 | 723 | 75....... | 26,237 | 2,476 |
| 33 ....... | 83,277 | 726 | 76....... | 23,761 | 2,431 |
| 34 ....... | 82,551 | 729 | 77....... | 21,330 | 2,369 |
| 35 ....... | 81,822 | 732 | 78....... | 18,961 | 2,291 |
| 36 ....... | 81,090 | 737 | 79....... | 16,670 | 2,196 |
| 37 ....... | 80,353 | 742 | 80....... | 14,474 | 2,091 |
| 38 ....... | 79,611 | 749 | 81....... | 12,383 | 1,964 |
| 39 ....... | 78,862 | 756 | 82....... | 10,419 | 1,816 |
| 40 ....... | 78,106 | 765 | 83....... | 8,603 | 1,648 |
| 41 ....... | 77,341 | 774 | 84....... | 6,955 | 1,470 |
| 42 ....... | 76,567 | 785 | 85....... | 5,485 | 1,292 |
| 43 ....... | 75,782 | 797 | 86....... | 4,193 | 1,114 |
| 44 ....... | 74,985 | 812 | 87....... | 3,079 | 933 |
| 45 ....... | 74,173 | 828 | 88....... | 2,146 | 744 |
| 46 ....... | 73,345 | 848 | 89....... | 1,402 | 555 |
| 47 ....... | 72,497 | 870 | 90....... | 847 | 385 |
| 48 ....... | 71,627 | 896 | 91....... | 462 | 246 |
| 49 ....... | 70,731 | 927 | 92....... | 216 | 137 |
| 50 ....... | 69,804 | 962 | 93....... | 79 | 58 |
| 51 ....... | 68,842 | 1,001 | 94....... | 21 | 18 |
| 52 ....... | 67,841 | 1,044 | 95....... | 3 | 3 |

